# Family Social Support and Weight-Related Behaviors of School-Age Children: An Exploratory Analysis

**DOI:** 10.3390/ijerph19148501

**Published:** 2022-07-12

**Authors:** Colleen L. Delaney, Carol Byrd-Bredbenner

**Affiliations:** Department of Nutritional Sciences, Rutgers University, New Brunswick, NJ 08901, USA; cld142@sebs.rutgers.edu

**Keywords:** family support, social support, children, physical activity, diet, sleep, children’s health, child wellbeing, evidence-informed practice

## Abstract

Families are a key provider of support that may encourage positive weight-related behaviors. Yet little is known about the relation of family support to children’s performance of weight-related behaviors. Mothers (N = 524) who completed an online survey were categorized as having low, moderate, or high family support for fruit/vegetable intake, breakfast intake, limiting sugar-sweetened beverage intake, physical activity, limiting sedentary screentime, and sleep. ANOVA revealed that children in families with high support for breakfast ate this meal significantly more often. Additionally, children in families with low support for limiting sugar-sweetened beverages had significantly greater intake. Surprisingly, families with moderate support for physical activity and sleep tended to have children with lower physical activity level, sleep duration, and sleep quality, and fewer days/week with set bedtimes than those with low and high support. Binomial logistic regression revealed that high family support for eating breakfast, limiting sugar-sweetened beverages, and getting sufficient sleep had greater odds of meeting recommendations for these behaviors. Findings suggest that greater family support for healthy weight-related behaviors tends to be associated with children’s performance of these behaviors. Future interventions should further examine the impact of different types of family support on weight-related behaviors to better understand this complex interplay.

## 1. Introduction

Childhood obesity is an ongoing epidemic in the U.S., affecting about 20% of children aged 6 to 11 as of 2018 [1]. Childhood obesity has serious health implications, including sleep apnea, non-alcoholic fatty liver disease, cardiovascular disease, dyslipidemia, metabolic syndrome, type 2 diabetes, psychosocial issues (e.g., low self-esteem, high risk of anxiety), early onset of thelarche and menarche, hyperandrogenism, and polycystic ovary syndrome [2]. Additionally, childhood obesity is associated with increased risk of adult obesity, which is related to poor health throughout adulthood and increased premature mortality [2]. Numerous modifiable factors increase obesity risk, including inadequate intake of fruits/vegetables, irregular breakfast consumption, excessive intake of sugar-sweetened beverages, limited physical activity, frequent sedentary activity, and inadequate sleep duration [3,4,5,6,7,8]

Parents play a pivotal role in mitigating obesity risk for children [9,10]. Parents primarily influence their children’s behaviors through role modeling and reinforcement of healthy behaviors [9,10]. Additionally, parents can influence children’s behavior by creating home environments that are more conducive to engaging in healthy behaviors (e.g., greater availability of fruits and vegetables in the home leads to greater consumption) [9,10].

Family support is another influence affecting children’s performance of behaviors, and ultimately their health status [11,12]. For example, families provide economic and material support that affects access to resources such as food, housing, and medical care. Families also give human support through teaching children knowledge and skills related to an array of topics, including health behaviors. This support may include passing along knowledge about the components of a healthy diet and teaching skills needed to select and prepare healthy meals. Families also provide social support, which includes social relationships, values, attitudes, and norms shared by the family [13]. Families may express positive social support through setting expectations for performing certain behaviors (e.g., going to bed at a certain time to ensure everyone gets enough sleep), encouraging each other to engage in behaviors (e.g., providing toys and sports equipment that promote active playtime, speaking positively about the importance of eating breakfast daily), and providing supportive environments that facilitate healthy behaviors (e.g., stocking home pantries with fruits and vegetables) and limit unhealthy behaviors (e.g., setting time limits on use of sedentary electronic media) [14]. Family may express negative social support for a behavior by not setting expectations or limiting access to environmental supports as well as by discouraging or resisting performance of a behavior. When positive family support is limited and/or negative family support prevails, access to factors supportive of good health typically is compromised.

Research suggests that greater family support for healthy behaviors has the potential for positively influencing the health-related behaviors of children—with the opposite also being true [14,15,16,17]. For example, a systematic literature review reported that siblings affect each other’s eating behaviors, often in negative ways, such as teasing each other about eating healthy foods [18,19]. Additionally, parents affect children’s behaviors in a variety of ways, such as responding to children’s resistance to eating foods they do not enjoy by pressuring them to eat [20]. Most studies investigate parental influences on children’s behavior with few examining the influence of siblings on each other or children on parents—that is, the family as a whole [18]. Indeed, an examination of the influence of the family as a unit is nearly absent from the literature [16]. Thus, the purpose of this exploratory secondary analysis was to address this gap by investigating the associations between family social support for key weight-related behaviors (i.e., eating, physical activity, sleep) and children’s performance of those behaviors.

## 2. Materials and Methods

This study was approved by the Institutional Review Board at the authors’ university (protocol number: Pro2020001192). All participants gave informed consent electronically by clicking an “agree” button prior to completing the only survey instrument.

### 2.1. Sample

English-speaking mothers between the ages of 24 and 54 years, with at least one child between the ages of 6 and 11 years, and who were the primary household food gatekeeper (i.e., made all or most decisions about foods purchased and prepared in the home) were recruited to complete the online “**H**ome **O**besogenicity **M**easure of **E**nvironment**S**—**F**amilies with **S**chool-**A**ge **K**ids-2” (HOMES-2) survey [21]. Mothers were studied because, compared to fathers, they have a greater burden of handling child care responsibilities (i.e., managing children’s schedules/activities, caring for children when they are sick, playing with or doing activities with children, disciplining children) as well as handling household chores and responsibilities whether or not they are employed outside the home. As a result, they were judged to be more accurate informants of child and family behaviors than fathers [22]. All participants were recruited from a research panel curated by Dynata, an online survey research company. Participants received modest compensation averaging about USD 10, which was awarded in the form of cash, points, prizes, or charitable donations with the specific amount determined by a proprietary algorithm that considers factors such as survey length, sample eligibility criteria, and length of time on the research panel.

### 2.2. Instruments

The HOMES-2 survey assessed weight-related aspects of households with school age children, including family support for engaging in behaviors supportive of healthy body weights, as well as weight-related behaviors. The development of this survey followed a carefully constructed and executed plan designed to incorporate best practices for questionnaire development to ensure assessments were valid and reliable; details are reported elsewhere [21].

The secondary analysis reported here includes sociodemographic characteristics of the family (e.g., maternal age, race/ethnicity, and education level; number of parents in the household; and region of residence in the United States). This analysis also included assessments of family social support for weight-related behaviors and children’s weight-related behaviors. As shown in Table 1, the Family Social Support for Healthy Behaviors Scale included 5 items focused on negative family social support (i.e., complaints about performing a behavior) that were answered using a 5-point Likert-type agreement scale. Answer choices ranged from strongly agree to strongly disagree, scored 1 to 5, respectively. Thus, higher scores indicate greater positive family support for the behavior. The negative phrasing is used in this scale because during scale development, which included iterative qualitative cognitive testing and quantitative pilot testing with a sample having the same characteristics of this study sample but not included in this sample, indicated that complaints were actions that were memorable, more likely to occur than compliments, and salient to mothers. Scale items focused on eating (3 items: fruits/vegetables, breakfast, sugar-sweetened beverages), physical activity (1 item), and sleep (1 item). The Cronbach-alpha coefficient for this scale was 0.87, indicating a high degree of internal consistency [23,24].

Mothers were asked to report data for one of their children aged 6 to 11 years. In families with more than one child in this age group, mothers were instructed to select the child born closest to a randomly selected date and time. Children’s assessed weight-related behaviors were analogous to the family social support variables. These included the Block Fruit/Vegetable screener to determine the number of servings of fruits and vegetables children ate each day [25,26,27]. Children’s breakfast frequency was equal to the number of days per week children regularly ate breakfast [21]. The HOMES Sugar-Sweetened Beverages Frequency was used to estimate children’s total daily servings of sugar-sweetened beverages (i.e., soft drinks, fruit and other sweetened drinks, sweetened coffee, energy drinks, sports drinks, smoothie drinks) [21,28,29]. Children’s physical activity was assessed using the Streamlined, Enhanced Self-Report Physical Activity Measure which has a score range of 0 to 42 [30,31,32] as well as time spent in sedentary screentime activities daily. Pittsburgh Sleep Quality Index components were used to assess sleep duration (hours/night) and sleep quality (5-point scale ranging from very bad to very good) [33,34]. In addition, total nights per week children had a set bedtime was reported.

### 2.3. Data Analysis

Analyses were conducted with SPSS software version 28 (IBM Corporation, Chicago, IL, USA). Descriptive statistics (e.g., means, standard deviations) were calculated to describe participant sociodemographic characteristics. Analysis of variance (ANOVA) and Tukey post hoc tests were used to compare children’s weight-related behaviors by level of family social support for the analogous item from the Family Social Support Scale. Participants with scores of ≤2, 3, and ≥4 were placed in the low, medium, and high levels of family social support for the specific behavior, respectively. Effect size for all significant differences was calculated as partial eta squared, with thresholds for small, medium, and large effect size at 0.01, 0.06, and 0.14, respectively [35]. The probability level was set at *p* < 0.05.

Binomial logistic regression models were used to estimate the odds ratio for meeting the recommendation for each weight-related behavior (model outcome) between those with a low and high level of family social support (primary predictor) for the behavior. Children of participants were categorized dichotomously as meeting or not meeting the recommendations for each of the weight-related behaviors. Specifically, the recommended fruit and vegetable intake was the age–sex specific midpoint of the range from MyPlate recommendations (i.e., 3.5 cups for children aged 5 to 8, 4 cups for girls aged 9 to 11, and 4.5 for boys aged 9 to 11) [36,37]. The breakfast intake recommendation was defined as eating breakfast daily [38]. The sugar-sweetened beverage intake recommendation was set at 0 drinks per day [39]. The physical activity level recommendation was set at ≥67% of the highest score possible on the Streamlined, Enhanced Self-Report Physical Activity Measure, which equates to about 2 h of activity daily [30,40]. Sedentary screentime behavior recommendation was ≤2 h/day [41]. Regarding sleep behaviors, the minimum sleep recommended for the 6 to 13 year age group (i.e., ≥9 h/night and <11 h/night) set by the National Sleep Foundation was used [42]. Recommended sleep quality was set at a rating of good or better [43,44]. Bedtime recommendation was defined as having a set bedtime daily [44].

## 3. Results

Demographic characteristics of the sample are shown in Table 2. Mothers averaged about 38 years of age and had approximately two children per household. Mothers were predominately White, Hispanic, and Black. Most mothers had at least some college education. Mothers predominately lived in dual parent households and were fairly evenly dispersed across the regions of the United States.

As shown in Table 3, ANOVA revealed that, regardless of family support level for fruits/vegetables, children had similar intakes equaling about 4 to 5 servings daily. Children in families with high support for breakfast ate this meal nearly every day, a rate that was significantly more often than those with less support for breakfast who ate breakfast about 5 days per week. Children had a quarter of a serving or less of sugar-sweetened beverages daily, with those in families giving low support for limiting these drinks having significantly smaller amounts than children in families with higher levels of family support.

Children in all families had low levels of physical activity, achieving about 50% or less of the total possible points on this scale. Children in families with medium support for physical activity had significantly lower physical activity levels than both of the other groups. Sedentary screentime equaled about 4 h per day and did not differ by level of family social support for physical activity.

When grouped by level of family social support for sleep, children in families giving moderate support for sleep got significantly less sleep than children with low and high family support for sleep. All children had good to very good sleep quality; however, children with a high level of family social support had significantly better sleep quality than those with less family support. In addition, children in families with high support for sleep had a set bedtime significantly more days per week than those with less support.

Table 4 describes the odds of children meeting recommendations for each weight-related behavior by family social support level (low vs. high) for the behavior. Family support for fruit and vegetable intake did not significantly impact the odds of meeting age and sex-based recommendations for child fruit and vegetable intake. However, the odds of meeting the recommendation for other weight-related behaviors increased when family support for the behavior was high, although not all associations were significant. For eating behaviors, those with high family support for breakfast and limiting sugar sweetened beverages had an increased odds of meeting the recommendations for these respective behaviors (OR = 6.62, OR = 4.58, respectively). Level of family support for physical activity was not significantly associated with increased odds of meeting recommendations for physical activity or sedentary behavior. Regarding sleep behaviors, level of family support for sleep did not significantly increase the odds of meeting recommendations for sleep duration or quality; however, those with high family support for sleep had significantly increased odds for having the recommended daily set bedtime for children (OR = 1.81).

## 4. Discussion

This study examined the relationship between family support for healthy weight-related behaviors and performance of these behaviors by children aged 6 to 11 years. Findings indicate that children in families with high support for health-related behaviors tended to have healthier weight-related behaviors. That is, these children tended to have more frequent breakfast intake, lower sugar-sweetened beverage intake, greater physical activity, improved sleep duration and quality, and a more frequent set bedtime. Additionally, binomial regressions found that high family support tended to increase the odds of meeting the recommendations for weight-related behaviors, with the odds being significant for breakfast, limiting sugar-sweetened beverage intake, and daily bedtime.

National data as well as numerous other studies have consistently shown that children are not meeting fruit and vegetable recommendations [39,45,46,47,48,49,50,51]. In contrast, most children in this study fell within the age and gender recommendations for fruit and vegetable intake, with 84% of the sample meeting at least the minimum MyPlate recommendations. It is not clear why these differences occurred. It may be that the public health messages promoting fruit and vegetable intake have penetrated this audience, leading to healthier intakes. Alternately, parents may have over-reported children’s fruit and vegetable consumption due to inaccurate estimations of intakes or, as seen in other studies, parents may have overestimated the healthfulness of their children’s diets, despite children not meeting recommendations [50,52]. Another possible explanation is that this study’s instrument aggregated fruits and vegetables—separating them may have revealed a differential effect for fruit vs. vegetables, which warrants further investigation.

The lack of relationship between family support level and fruit and vegetable intake supports work by Neumark-Sztainer et al., who reported no correlation between fruit and vegetable intake and social support for healthy eating (i.e., parental support for healthy eating (“My mother cares about healthy food.”, “My mother encourages me to eat healthy food.”) and peer support for healthy eating (“Many of my friends care about healthy food.”)) [53]. However, Metcalfe et al., who examined family support in terms of family food involvement (e.g., “I involve my child in planning family meals”) reported a significant relationship between food involvement and increased fruit and vegetable intake [54]. These contrasting findings are likely due to the difference in how family support was conceptualized—as perceptions vs. behaviors—and indicates the importance of clearly describing assessments used and comparing results generated by differing assessments in future research.

Breakfast is an important factor in energy balance and dietary regulation, as well as improved school performance and overall health [38,55,56]. Breakfast skipping increases with age in children and is positively associated with overweight and obesity, cardiometabolic risk, and poor diet [38,55,57]. The U.S. Dietary Guidelines recognize that breakfast is an important component to a healthy lifestyle for everyone [39]. Yet, national data show that about 15% of children aged 6 to 11 skip breakfast, with the prevalence of breakfast skipping increasing to about a third of the teenage population [58]. Like other research reporting higher rates of breakfast skipping, [59,60] about a third of the children in this study did not meet the daily breakfast recommendation. Family support for breakfast eating was clearly related in this study, with children having high family support eating breakfast significantly more days per week than those with low family support. Future research should aim to elucidate the factors contributing to greater family support for this meal.

Limiting sugar-sweetened beverage intake is important to achieving a healthy dietary pattern as these drinks often displace nutrient dense drinks, such as milk, 100% juice, and water [39,61]. Additionally, excess sugar has been associated with an increased risk for poor health, including dental carries, cardiovascular disease, dyslipidemia, insulin resistance, type 2 diabetes mellitus, and fatty liver disease [62,63,64,65,66,67,68,69,70,71]. Therefore, the dietary guidelines have recommended limiting added sugar to 10% of total energy intake, yet national data indicates that about 80% of young children are exceeding this recommendation [39], with about 15 to 25% of total added sugars attributed to beverages, increasing to about 32% of added intake in adolescence [39]. Similarly, many studies have reported that children are exceeding recommendations for limiting added sugar, particularly due to the intake of sugary drinks [72,73,74]. In contrast, children in this study had few sugar-sweetened beverages, averaging less than one-half serving per day. It may be that parents in this study under-reported child intake due to lack of knowledge of children’s consumption at school (e.g., sugar-sweetened low-fat milk) or after-school activities (e.g., sports drinks) [75]. Although children had low intake of sugar-sweetened beverages, a clear relationship was seen between intake and family support for limiting sugar sweetened beverages; children in families with low support had significantly more sugar-sweetened beverages than those in families with high support. Future research should aim to elucidate factors contributing to greater family support for limiting sugar-sweetened beverage intake.

Physical activity is important in childhood as it provides a foundational skill for lifelong health and wellbeing [39,76]. School-aged children should get at least 60 min of moderate-to-vigorous physical activity daily, in addition to about 3 days per week of muscle-strengthening and bone-strengthening activities [39,76]. Children who are physically active have improved health outcomes, including cardiovascular health, improved cognition, and a reduction in depression [76]. Despite the many benefits of physical activity, children in the U.S. are largely not meeting recommendations, with only about 20% of children meeting physical activity guidelines [77,78,79]. Similarly, studies have found that older children and teens are not meeting physical activity guidelines [5,80]. Children in this study had low-moderate physical activity. Additionally, physical activity was highest in children with low and high family support for physical activity. In a study conducted by Medd et al., intent to support did not always indicate enacted support—that is, because parents are aware of the importance of a behavior, they intend to support their child but do not always act on that support [81]. Future studies should examine other forms of support that elucidate what types of family support lead to enacted support versus intended support of physical activity as well as discern why children with low and high support had similar activity levels. It may that moderate support families are not accurately reporting screentime and physical activity, as both were lower compared to other families; it would be expected that screentime would replace physical activity, but that does not appear to be the case in this study. Additionally, families may be overestimating physical activity as they assume their children are physically active while at school. Objective measurement of physical activity in future studies could help clarify this finding.

Increased sedentary behavior can displace physical activity and sleep, leading to poor health outcomes [39,41,76]. The American Academy of Pediatrics recognizes that there are potential benefits to screentime—including exposure to new ideas and increased opportunities for social support—while acknowledging its potential risks on health; therefore, it is recommended that children spend 2 h or less engaged in screentime daily [41]. Despite this, national data indicate that about 60% of children continue to not meet screentime recommendations [41,77,78].

Children in this study exceeded screentime recommendations, spending about 3.5 to over 4 h engaged in screentime daily; however, family support for limiting screentime was not found to be associated with increased screentime in children. It may be that sedentary media is so ubiquitous in our current culture that, despite support for limiting screentime, it is difficult for parents to bring the implementation of desired limits to fruition.

The National Sleep Foundation recommends that children aged 6 to 13 get between 9 and 11 h of sleep nightly [42]. Adequate sleep is associated with better outcomes, such as improved attention, behavior, learning, quality of life, and mental and physical health in children, whereas inadequate sleep is associated with learning problems, hypertension, obesity, diabetes, and depression [82]. National data indicate that most children are meeting sleep recommendations [78]; in contrast, half of the children in this study did not meet sleep recommendations. Another important aspect of sleep is quality; poor sleep quality is associated with adverse health outcomes, including type 2 diabetes mellitus, obesity, psychosocial issues, cardiovascular disease, and reduced quality of life [83,84,85]. Between 25 and 50% of children in the U.S. have sleep problems that contribute to poor sleep quality and about a third to half of parents report their children have poor sleep quality; however, parents in this study reported that, on average, children had good to very good sleep quality [86,87,88]. Consistent bedtimes and bedtime routines are associated with improved sleep duration and quality. Despite these benefits, national data suggest that children have inconsistent bedtimes; this discrepancy was also seen in this study, with half of the children not having a bedtime nightly [89].

Surprisingly, children in families with both low and high support had greater sleep duration, better sleep quality, and more consistent bedtimes than children in families with moderate support. Further analysis revealed that about half of all children in the low and high family support groups met age-based sleep recommendations, whereas only 40% of those in the moderate family support group met age-based sleep recommendations. It may be that length of sleep is linked to quality and having a routine bedtime; that is, poor sleep quality may lead to shorter sleep, and inconsistent and erratic bedtimes may also contribute to shorter sleep and poorer sleep quality [44,90,91]. Additionally, a clear trend was noted for bedtime, where families with high support of sleep had an increased likelihood of meeting daily bedtime recommendations compared to families with low support. It is likely that families with high support tended to have home environments that fostered better sleep quality and duration and that promoted greater consistency in bedtime routine. Future studies should be conducted to further explore the relationship between family support and sleep quality.

This study is, to the best of the authors’ knowledge, one of the first to examine the relationship between family support for specific weight-related behaviors and children’s performance of these behaviors. This study has many strengths, including a large sample size with representation across the United States. In addition, it used valid, reliable scales to assess family support and children’s behaviors. This study is limited in that it is a secondary analysis of data collected for another purpose which constrained the assessment of the concept of family social support to consider only family complaints as indicative of support or lack of support for a behavior. However, complaints tend to be memorable, and items stated in the converse (e.g., My family said they enjoyed eating fruits and vegetables) may increase social desirability risk. Finally, all data were self-reported by mothers who may not have been fully informed of children’s dietary and physical activity behaviors while at school, and the cross-sectional design does not allow for determination of causation.

Despite the study limitations, the findings are unique in that they examine the relationship between behavior-specific family support and its matched weight-related behavior. It is important to consider the impact of behavior-specific family support. A family may exhibit a variety of types of support—support may be functional (e.g., emotional, informational, tangible), structural (e.g., family composition), or combined [92]. To apply this concept to this study, a family may agree that a behavior is important and have the intent to support that behavior but lack the capabilities due to constraints, such as a lack of social support from their children or spouse or a home environment that does not ultimately support that behavior [92]. Additionally, support is behavior specific: a family may not have the same outcome expectations or tangible support for different behaviors and therefore would have varying levels of support for the behaviors [92].

## 5. Conclusions

In conclusion, the findings of this study indicate that family social support is positively related to the performance of key weight-related behaviors by children. This finding points to the importance of stressing the value of family support to participants in health behavior change interventions as well as providing opportunities to build participant skills for helping family members support each other in their efforts of practicing healthy weight-related behaviors. Future studies should further examine the effect of different behavior-specific types of social support (i.e., structural, functional) on weight-related behaviors, expand the audience to younger children as well as adolescents to determine the relative importance of family support at different developmental stages, and investigate how factors in the broader physical and social environment (e.g., neighborhoods, schools, community) may moderate support provided by the family. In addition, collecting data from children themselves would be useful to fill in any gaps in parent knowledge of children’s dietary and physical activity behaviors while at school.

## Figures and Tables

**Table 1:** Family Social Support for Healthy Behaviors Scale ^1^.

Family Support for Behavior	Survey Item
**Eating**	
Fruits/Vegetables	In the last 2 weeks, my family complained about eating fruits and vegetables.
Breakfast	In the last 2 weeks, my family complained about eating breakfast.
Sugar-Sweetened Beverages	In the last 2 weeks, my family complained about having to limit sugary drinks.
**Physical Activity**	In the last 2 weeks, my family complained about having to be physically active for a total of at least 60 min every day.
**Sleep**	In the last 2 weeks, my school-age kids complained about having to go to bed on time.

^1^ Five-point Likert agreement scale was used where 1 = strongly agree and 5 = strongly disagree. Higher scores for each survey item indicate greater family support for the behavior.

**Table 2:** Sociodemographic characteristics (N = 524).

Characteristic	Mean ± SD or N (%) (95% CI *)
**Maternal Age**	37.57 ± 5.81 (37.08, 38.08)
**Number of Children in Household**	2.20 ± 1.05 (2.11, 2.29)
**Race/Ethnicity**	
White	185 (35.3%)
Non-white	339 (64.7%)
Hispanic, Latino, or Spanish	130 (24.8%)
Black or African American	103 (23.9%)
American Indian or Alaskan Native	4 (0.8%)
Asian Indian	24 (4.6%)
Other Asian (e.g., Japanese, Chinese, Korean)	48 (9.2%)
Other	30 (5.7%)
**Education Level**	
High school or less	92 (17.6%)
Some college	182 (34.7%)
College graduate or higher	250 (47.7%)
**Parents in Household**	
1 Parent	111 (21.2%)
2 Parents	413 (78.8%)
**U.S. Region of Residence**	
Eastern	115 (21.9%)
Midwestern	83 (34.7%)
Southern	182 (34.7%)
Western	144 (27.5%)

* CI = Confidence Interval.

**Table 3:** Relationships among family social support for children’s weight-related behaviors (N = 524).

Behavior	Family Social Support Level			*F* *df* = 2521 **	ANOVA † *p*	Partial Eta Squared
	Low Mean ± SD (95% CI *)	Medium Mean ± SD (95% CI *)	High Mean ± SD (95% CI *)			
**Eating**						
Fruits/Vegetables (servings/day)	n = 82	n = 98	n = 344			
	4.73 ± 1.62 (4.38, 5.09)	4.52 ± 1.68 (4.18, 4.86)	4.35 ± 1.59 (4.19, 4.52)	1.981	0.139	0.008
Breakfast (days/week)	n = 91	n = 66	n = 367			
	4.77 ± 2.22 (4.31, 5.23)	4.62 ± 2.48 (4.01, 5.23)	6.37 ± 1.45 (6.22, 6.52)	49.303	<0.001 ^BC^	0.159
Limiting Sugar-Sweetened Beverage (servings/day)	n = 143	n = 80	n = 301			
	0.26 ± 0.32 (0.21, 0.31)	0.17 ± 0.19 (0.13, 0.22)	0.11 ± 0.19 (0.09, 0.14)	19.373	<0.001 ^AB^	0.069
**Physical Activity**	n = 145	n = 92	n = 287			
Physical Activity Level ^1^	20.94 ± 11.37 (19.08, 22.81)	16.77 ± 10.87 (14.52, 19.02)	22.57 ± 12.80 (21.08, 24.06)	8.024	<0.001 ^AC^	0.030
Sedentary Screentime Behavior (hours/day)	4.08 ± 2.65 (3.64, 4.51)	3.63 ± 2.63 (3.08, 4.17)	4.12 ± 2.81 (3.80, 4.45)	1.198	0.303	0.005
**Sleep**	n = 196	n = 80	n = 248			
Sleep Duration (hours/day)	8.86 ± 1.38 (8.67, 9.06)	8.15 ± 1.87 (7.73, 8.57)	8.85 ± 1.22 (8.70, 9.01)	8.695	<0.001 ^AC^	0.032
Sleep Quality ^2^	4.38 ± 0.63 (4.29, 4.47)	4.29 ± 0.83 (4.10, 4.47)	4.50 ± 0.64 (4.42, 4.58)	3.891	0.021 ^C^	0.015
Bedtime (days/week)	5.89 ± 2.42 (5.55, 6.23)	5.66 ± 2.40 (5.13, 6.20)	6.60 ± 2.14 (6.33, 6.87)	7.763	<0.001 ^BC^	0.029

* CI = Confidence interval. ** *df* = Degrees of freedom. † Analysis of variance (ANOVA). Superscript capital letters indicate significant (*p* < 0.05) Tukey post hoc tests results: ^A^ low vs. medium, ^B^ low vs. high, ^C^ medium vs. high. ^1^ Possible score range = 0 to 42; higher scores indicate more physical activity [30]. ^2^ Five-point Likert-type response scale, where 1 = very bad, bad, OK, good, and 5 = very good. Higher scores indicate better sleep quality [33].

**Table 4:** Binomial regression: odds ratios of children meeting recommendations for health behaviors and high level of family support (N = 524).

Behavior	Family Social Support	Odds Ratio (95% CI *)	*p*
MyPlate Fruit/Vegetable Recommended Servings/Day ^1^	Fruits/Vegetables	0.73 (0.43, 1.22)	0.229
Breakfast Daily	Breakfast	6.62 (4.03, 10.87)	<0.010
Sugar-Sweetened Beverage 0 servings/day	Limiting Sugar-Sweetened Beverage	4.58 (2.41, 8.70)	<0.001
Physical Activity ≥ 67% score ^2^	Physical Activity	1.32 (0.86, 2.03)	0.198
Sedentary Screentime ≤ 2 h/day	Physical Activity	1.161 (0.75, 1.81)	0.509
Sleep (9 to 11 h/day)	Sleep	1.07 (0.73, 1.55)	0.736
Sleep Quality (Good or Better)	Sleep	1.92 (0.87, 4.23)	0.108
Bedtime Daily	Sleep	1.81 (1.24, 2.64)	0.002

* CI = Confidence interval. ^1^ MyPlate recommended midpoint of range of daily servings of fruits and vegetables: 3.5 for children aged 5–8; 4 for girls aged 9–11; and 4.5 for boys aged 9–11 [36,37]. ^2^ Possible score range = 0 to 42; recommendation represents achieving 67% of goal [30].

## Data Availability

Data sharing is not applicable to this article. No new data were created or analyzed in this study.

## References

[1] Centers for Disease Control and Prevention Childhood Obesity Facts. https://www.cdc.gov/obesity/data/childhood.html.

[2] Yanovski J. (2015). Pediatric obesity. An introduction. Appetite.

[3] Anderson S.E., Whitaker R.C. (2010). Household routines and obesity in US preschool-aged children. Pediatrics.

[4] Whitaker R.C., Wright J.A., Pepe M.S., Seidel K.D., Dietz W.H. (1997). Predicting obesity in young adulthood from childhood and parental obesity. N. Engl. J. Med..

[5] Beck J., De Witt P., McNally J., Siegfried S., Hill J., Stroebele-Benschop N. (2015). Predictors of meeting physical activity and fruit and vegetable recommendations in 9–11-year-old children. Health Educ. J..

[6] Liao J., Cao C., Hur J., Cohen J., Chen W., Zong X., Colditz G., Yang L., Stamatakis E., Cao Y. (2021). Association of sedentary patterns with body fat distribution among US children and adolescents: A population-based study. Int. J. Obes..

[7] Narcisse M., Long C., Felix H., Howie E., Purvis R., McElfish P. (2019). Adherence to sleep guidelines reduces risk of overweight/obesity in addition to 8-5-2-1-0 guidelines among a large sample of adolescents in the United States. Sleep Health.

[8] Verduci E., DiProfia E., Fiore G., Zuccotti G. (2022). Integrated approaches to combating childhood obesity. Ann. Nutr. Metab..

[9] Rosenkranz R., Dzewaltowski D. (2008). Model of the home food environment pertaining to childhood obesity. Nutr. Rev..

[10] Guruber K., Haldeman L. (2009). Using the family to combat childhood and adult obesity. Prev. Chronic Dis..

[11] Zeng S., Hu X., Zhao H., Stone-MacDonald A.K. (2020). Examining the relationships of parental stress, family support and family quality of life: A structural equation modeling approach. Res. Dev. Disabil..

[12] Kyzar K., Turnbull A., Summers J., Gomez V. (2012). The relationship of family support to family outcomes: A synthesis of key findings from research on severe disability. Res. Pract. Persons Servere Disabl..

[13] Quick V., Delaney C., Eck K., Byrd-Bredbenner C. (2021). Family social capital: Links to weight-related and parenting behaviors of mothers with young children. Nutrients.

[14] Rotman S., Fowler L., Ray M., Stein R., Hayes J., Kolko R., Balantekin K., Engel A., Saelens B., Welch R. (2020). Family encouragement of healthy eating predicts child dietary intake and weight loss in family-based behavioral weight-loss treatment. Child. Obes..

[15] Heredia N., Ranjit N., Warren J., Evans A. (2016). Association of parental social support with energy balance-related behaviors in low income and ethnically diverse children: A cross-sectional study. BMC Public Health.

[16] Yee A., Lwin M., Ho S. (2017). The influence of parental practices on child promotive and preventive food consumption behaviors: A systematic review and meta-analysis. Int. J. Behav. Nutr. Phys. Act..

[17] Lopez N., Ayala G., Corder K., Eisenberg C., Zive M., Wood C., Elder J. (2012). Parent support and parent mediated behaviors are associated with children’s sugary beverage consumption. J. Acad. Nutr. Diet..

[18] Ragelienė T., Grønhøj A. (2020). The influence of peers’ and siblings’ on children’s and adolescents’ healthy eating behavior. A systematic literature review. Appetite.

[19] Povey R., Cowap L., Gratton L. (2016). “They said I’m a square for eating them”. Children’s beliefs about fruit and vegetables in England. Br. Food J..

[20] Farrow C.V., Galloway A.T., Fraser K. (2009). Sibling eating behaviours and differential child feeding practices reported by parents. Appetite.

[21] Byrd-Bredbenner C., Santiago E., Eck K., Delaney C., Quick V., Pozzoli A., Worobey J., Shelnutt K., Olfert M. (2022). HomeStyles-2: Randomized controlled trial protocol for a web-based obesity prevention program for families with children in middle childhood. Contemp. Clin. Trials.

[22] Pew Research Center Raising Kids and Running a Household: How Working Parents Share the Load. https://www.pewresearch.org/social-trends/2015/11/04/raising-kids-and-running-a-household-how-working-parents-share-the-load/.

[23] Nunnally J. (1978). Psychometric Theory.

[24] Tavakol M., Dennick R. (2011). Making sense of Cronbach’s alpha. Int. J. Med. Educ..

[25] Block G., Gillespie C., Rosenbaum E., Jenson C. (2000). A rapid food screener to assess fat and fruit and vegetable intake. Am. J. Prev. Med..

[26] Block G., Hartman A., Naughton D. (1990). A reduced dietary questionnaire: Development and validation. Epidemiology.

[27] Block G., Thompson F., Hartman A., Larkin F., Guire K. (1992). Comparison of two dietary questionnaires validated against multiple dietary records collected during a 1-year period. J. Am. Diet. Assoc..

[28] Byrd-Bredbenner C., Martin-Biggers J., Koenings M., Quick V., Hongu N., Worobey J. (2017). HomeStyles, A web-based childhood obesity prevention program for families with preschool children: Protocol for a randomized controlled trial. JMIR Res. Protoc..

[29] Santiago E. (2021). Relationships among Maternal Employment and Weight-Related Cognitions Behaviors, and Home Environments of Mothers and Their School-Age Children.

[30] Quick V., Byrd-Bredbenner C., Shoff S., White A., Lohse B., Horacek T., Kattleman K., Phillips B., Hoerr S., Greene G. (2016). A streamlined, enhanced self-report physical activity measure for young adults. Int. J. Health Promot. Educ..

[31] Lee P.H., Macfarlane D.J., Lam T.H., Stewart S.M. (2011). Validity of the International Physical Activity Questionnaire Short Form (IPAQ-SF): A systematic review. Int. J. Behav. Nutr. Phys. Act..

[32] Craig C.L., Marshall A.L., Sjöström M., Bauman A.E., Booth M.L., Ainsworth B.E., Pratt M., Ekelund U., Yngve A., Sallis J.F. (2003). International physical activity questionnaire: 12-country reliability and validity. Med. Sci. Sports Exerc..

[33] Buysse D., Reynolds C., Monk T., Berman S., Kupfer D. (1989). The Pittsburgh Sleep Qualtiy Index: A new instrument for psychiatric practice and research. Psychiatr. Res..

[34] Carpenter J., Andrykowski M. (1998). Psychometric evaluation of the Pittsburgh Sleep Qualtiy Index. J. Psychosom. Res..

[35] Watson P. Rule of Thumb on Magnitudes of Effect Size. https://imaging.mrc-cbu.cam.ac.uk/statswiki/FAQ/effectSize.

[36] MyPlate Vegetables: More about the Vegetable Group. https://www.myplate.gov/eat-healthy/vegetables.

[37] MyPlate Fruits: More about the Fruit Group. https://www.myplate.gov/eat-healthy/fruits.

[38] Rampersaud G.C., Pereira M.A., Girard B.L., Adams J., Metzl J.D. (2005). Breakfast habits, nutritional status, body weight, and academic performance in children and adolescents. J. Am. Diet. Assoc..

[39] U.S. Department of Agriculture, U.S. Department of Health and Human Services Dietary Guidelines for Americans, 2020–2025. DietaryGuidelines.gov.

[40] Centers for Disease Control and Prevention How Much Physical Activity do Children Need?. https://www.cdc.gov/physicalactivity/basics/children/index.htm.

[41] Hill D., Ameenuddin N., Reid Chassiakos Y., Cross C., Radesky J., Hutchinson J., Levine A., Boyd R., Mendelson R., Council on Communications and Media (2016). Media use in school-aged children and adolescents. Pediatrics.

[42] National Sleep Foundation How Much Sleep do you Really Need?. https://www.thensf.org/how-many-hours-of-sleep-do-you-really-need/.

[43] Suni E., Singh A. How Much Sleep do We Really Need?. https://www.sleepfoundation.org/how-sleep-works/how-much-sleep-do-we-really-need.

[44] National Center for Chronic Disease Prevention and Health Promotion, Centers for Disease Control and Prevention (2021). Do Your Children Get Enough Sleep?.

[45] Lorson B.A., Melgar-Quinonez H.R., Taylor C.A. (2009). Correlates of fruit and vegetable intakes in US children. J. Am. Diet. Assoc..

[46] Kim S.A., Moore L.V., Galuska D., Wright A.P., Harris D., Grummer-Strawn L.M., Merlo C.L., Nihiser A.J., Rhodes D.G. (2014). Vital signs: Fruit and vegetable intake among children—United States, 2003–2010. MMWR Morb. Mortal. Wkly. Rep..

[47] Trofholz A.C., Tate A.D., Draxten M.L., Rowley S.S., Schulte A.K., Neumark-Sztainer D., MacLehose R.F., Berge J.M. (2017). What’s being served for dinner? An exploratory investigation of the associations between the healthfulness of family meals and child dietary intake. J. Acad. Nutr. Diet..

[48] Brady L.M., Lindquist C.H., Herd S.L., Goran M.I. (2000). Comparison of children’s dietary intake patterns with US dietary guidelines. Br. J. Nutr..

[49] Haughton C.F., Wang M.L., Lemon S.C. (2016). Racial/ethnic disparities in meeting 5-2-1-0 recommendations among children and adolescents in the United States. J. Pediatr..

[50] Eliason J., Acciai F., DeWeese R.S., Vega-López S., Ohri-Vachaspati P. (2020). Children’s consumption patterns and their parent’s perception of a healthy diet. Nutrients.

[51] Kegler M.C., Hermstad A., Haardörfer R. (2021). Home food environment and associations with weight and diet among U.S. adults: A cross-sectional study. BMC Public Health.

[52] Briefel R.R., Deming D.M., Reidy K.C. (2015). Parents’ perceptions and adherence to children’s diet and activity recommendations: The 2008 Feeding Infants and Toddlers Study. Prev. Chronic Dis..

[53] Neumark-Sztainer D., Wall M., Perry C., Story M. (2003). Correlates of fruit and vegetable intake among adolescents. Findings from Project EAT. Prev. Med..

[54] Metcalfe J.J., Fiese B.H. (2018). Family food involvement is related to healthier dietary intake in preschool-aged children. Appetite.

[55] Ricotti R., Caputo M., Monzani A., Pigni S., Antoniotti V., Bellone S., Prodam F. (2021). Breakfast skipping, weight, cardiometabolic risk, and nutrition quality in children and adolescents: A systematic review of randomized controlled and intervention longitudinal trials. Nutrients.

[56] Gingras V., Rifas-Shiman S.L., Taveras E.M., Oken E., Hivert M.F. (2018). Dietary behaviors throughout childhood are associated with adiposity and estimated insulin resistance in early adolescence: A longitudinal study. Int. J. Behav. Nutr. Phys. Act..

[57] Ramsay S.A., Bloch T.D., Marriage B., Shriver L.H., Spees C.K., Taylor C.A. (2018). Skipping breakfast is associated with lower diet quality in young US children. Eur. J. Clin. Nutr..

[58] Terry A.L., Wambogo E., Ansai N., Ahluwalia N. (2020). Breakfast intake among children and adolescents: United States, 2015-2018. NCHS Data Brief..

[59] Mahoney C.R., Taylor H.A., Kanarek R.B., Samuel P. (2005). Effect of breakfast composition on cognitive processes in elementary school children. Physiol. Behav..

[60] Edwards J.U., Mauch L., Winkelman M.R. (2011). Relationship of nutrition and physical activity behaviors and fitness measures to academic performance for sixth graders in a midwest city school district. J. Sch. Health.

[61] Muth N.D., Dietz W.H., Magge S.N., Johnson R., Bolling C.F., Armstrong S.C., Haemer M.A., Rausch J., Rogers V., Abrams S. (2019). Public policies to reduce sugary drink consumption in children and adolescents. Pediatrics.

[62] Moynihan P., Petersen P.E. (2004). Diet, nutrition and the prevention of dental diseases. Public Health Nutr..

[63] Vos M.B., Kaar J.L., Welsh J.A., Van Horn L.V., Feig D.I., Anderson C.A.M., Patel M.J., Cruz Munos J., Krebs N.F., Xanthakos S.A. (2017). Added sugars and cardiovascular disease risk in children: A scientific statement from the American Heart Association. Circulation.

[64] Shah N.S., Leonard D., Finley C.E., Rodriguez F., Sarraju A., Barlow C.E., DeFina L.F., Willis B.L., Haskell W.L., Maron D.J. (2018). Dietary patterns and long-term survival: A retrospective study of healthy primary care patients. Am. J. Med..

[65] Wang J.W., Mark S., Henderson M., O’Loughlin J., Tremblay A., Wortman J., Paradis G., Gray-Donald K. (2013). Adiposity and glucose intolerance exacerbate components of metabolic syndrome in children consuming sugar-sweetened beverages: QUALITY cohort study. Pediatr. Obes..

[66] Welsh J.A., Sharma A., Cunningham S.A., Vos M.B. (2011). Consumption of added sugars and indicators of cardiovascular disease risk among US adolescents. Circulation.

[67] Malik V.S., Popkin B.M., Bray G.A., Després J.P., Willett W.C., Hu F.B. (2010). Sugar-sweetened beverages and risk of metabolic syndrome and type 2 diabetes: A meta-analysis. Diabetes Care.

[68] O’Sullivan T.A., Oddy W.H., Bremner A.P., Sherriff J.L., Ayonrinde O.T., Olynyk J.K., Beilin L.J., Mori T.A., Adams L.A. (2014). Lower fructose intake may help protect against development of nonalcoholic fatty liver in adolescents with obesity. J. Pediatr. Gastroenterol. Nutr..

[69] Chen L., Caballero B., Mitchell D.C., Loria C., Lin P.H., Champagne C.M., Elmer P.J., Ard J.D., Batch B.C., Anderson C.A. (2010). Reducing consumption of sugar-sweetened beverages is associated with reduced blood pressure: A prospective study among United States adults. Circulation.

[70] Perez-Pozo S.E., Schold J., Nakagawa T., Sánchez-Lozada L.G., Johnson R.J., Lillo J.L. (2010). Excessive fructose intake induces the features of metabolic syndrome in healthy adult men: Role of uric acid in the hypertensive response. Int. J. Obes..

[71] Lee A.K., Binongo J.N., Chowdhury R., Stein A.D., Gazmararian J.A., Vos M.B., Welsh J.A. (2014). Consumption of less than 10% of total energy from added sugars is associated with increasing HDL in females during adolescence: A longitudinal analysis. J. Am. Heart Assoc..

[72] Dai J., Soto M.J., Dunn C.G., Bleich S.N. (2021). Trends and patterns in sugar-sweetened beverage consumption among children and adults by race and/or ethnicity, 2003–2018. Public Health Nutr..

[73] Spruance L.A., Bennion N., Ghanadan G., Maddock J.E. (2021). An educational intervention for improving the snacks and beverages brought to youth sports in the USA. Int. J. Environ. Res. Public Health.

[74] Palakshappa D., Lenoir K., Brown C.L., Skelton J.A., Block J.P., Taveras E.M., Lewis K.H. (2020). Identifying geographic differences in children’s sugar-sweetened beverage and 100% fruit juice intake using health system data. Pediatr. Obes..

[75] Murray R., Bhatia J., Okamoto J., Allison M., Ancona R., Attisha E., De Pinto C., Holmes B., Council on School Health, Committee on Nutrition (2015). Snacks, sweetened beverages, added sugars, and schools. Pediatrics.

[76] U.S. Department of Health and Human Services (2018). Physical Activity Guidelines for Americans.

[77] Katzmarzyk P.T., Denstel K.D., Beals K., Bolling C., Wright C., Crouter S.E., McKenzie T.L., Pate R.R., Saelens B.E., Staiano A.E. (2016). Results from the United States of America’s 2016 report card on physical activity for children and youth. J. Phys. Act. Health.

[78] Friel C.P., Duran A.T., Shechter A., Diaz K.M. (2020). U.S. children meeting physical activity, screen time, and sleep guidelines. Am. J. Prev. Med..

[79] Division of Population Health, National Center for Chronic Disease Prevention and Health Promotion Physical Activity Facts. https://www.cdc.gov/healthyschools/physicalactivity/facts.htm.

[80] Guthold R., Stevens G.A., Riley L.M., Bull F.C. (2020). Global trends in insufficient physical activity among adolescents: A pooled analysis of 298 population-based surveys with 1·6 million participants. Lancet Child. Adolesc. Health.

[81] Medd E.R., Beauchamp M.R., Blanchard C.M., Carson V., Gardner B., Warburton D.E., Rhodes R.E. (2020). Family-based habit intervention to promote parent support for child physical activity in Canada: Protocol for a randomised trial. BMJ Open.

[82] Paruthi S., Brooks L.J., D’Ambrosio C., Hall W.A., Kotagal S., Lloyd R.M., Malow B.A., Maski K., Nichols C., Quan S.F. (2016). Consensus statement of the American Academy of Sleep Medicine on the recommended amount of sleep for healthy children: Methodology and discussion. J. Clin. Sleep Med..

[83] Medic G., Wille M., Hemels M.E. (2017). Short- and long-term health consequences of sleep disruption. Nat. Sci. Sleep.

[84] Knutson K.L., Ryden A.M., Mander B.A., Van Cauter E. (2006). Role of sleep duration and quality in the risk and severity of type 2 diabetes mellitus. Arch. Intern. Med..

[85] Tsai Y.W., Kann N.H., Tung T.H., Chao Y.J., Lin C.J., Chang K.C., Chang S.S., Chen J.Y. (2012). Impact of subjective sleep quality on glycemic control in type 2 diabetes mellitus. Fam. Pract..

[86] Bhargava S. (2011). Diagnosis and management of common sleep problems in children. Pediatr. Rev..

[87] Matricciani L., Fraysse F., Grobler A.C., Muller J., Wake M., Olds T. (2019). Sleep: Population epidemiology and concordance in Australian children aged 11-12 years and their parents. BMJ Open.

[88] Nugent C.N., Black L.I. (2016). Sleep Duration, Quality of Sleep, and Use of Sleep Medication, by Sex and Family Type, 2013-2014. NCHS Data Brief..

[89] Jackson D.B., Testa A., Semenza D.C. (2021). Sleep Duration, Bedtime Consistency, and School Readiness: Findings from the 2016 to 2018 National Survey of Children’s Health. J. Dev. Behav. Pediatr..

[90] Golley R.K., Maher C.A., Matricciani L., Olds T.S. (2013). Sleep duration or bedtime? Exploring the association between sleep timing behaviour, diet and BMI in children and adolescents. Int. J. Obes..

[91] Golem D.L., Martin-Biggers J.T., Koenings M.M., Davis K.F., Byrd-Bredbenner C. (2014). An integrative review of sleep for nutrition professionals. Adv. Nutr..

[92] Glanz K., Rimer B.K., Viswanath K. (2015). Health Behavior: Theory, Research and Practice.

